# Binge eating disorder recognition and stigma among an adult community sample

**DOI:** 10.1186/s40337-024-01162-1

**Published:** 2025-01-10

**Authors:** Marilou Côté, Marie-Pier Roy, Christopher Rodrigue, Catherine Bégin

**Affiliations:** 1 Département des Fondements et Pratiques en Éducation, Université Laval, 2320, rue des Bibliothèques, Quebec, G1V 0A6 Canada; 2 Centre Nutrition, Santé et Société (NUTRISS), Institut sur la Nutrition et les Aliments Fonctionnels, Université Laval, 2440, boulevard Hochelaga Québec, Quebec, G1V 0A6 Canada; 3 Centre d’expertise Poids, Image et Alimentation, Université Laval, 2440, boulevard Hochelaga Québec, Quebec, G1V 0A6 Canada; 4 École de Psychologie, Université Laval, 2325, rue des Bibliothèques, Quebec, G1V 0A6 Canada; 5 Restigouche Hospital Center - Forensic and Tertiary Youth Psychiatry Unit, 63 Gallant Drive, Campbellton, NB E3N 3G2 Canada

**Keywords:** Binge eating disorder, Knowledge, Mental health literacy, Stigma, Weight bias, Vignette study

## Abstract

**Background:**

Despite being the most prevalent eating disorder, Binge eating disorder (BED) remains largely unrecognized and lacks awareness among the general public, where it is also highly stigmatized. Common stigma surrounding BED includes the belief that individuals with this disorder are responsible for their condition and lack willpower and self-control. Research on BED recognition and stigma among lay adults is scarce. Enhancing public recognition of BED and reducing the stigma associated with it is crucial, as this could significantly improve access to treatment. The aim of the present study was to examine BED recognition and stigma within an adult community sample, and to identify associated respondent characteristics, including sociodemographic and psychosocial factors.

**Methods:**

A sample of 894 adults (88.6% women; M_age_ = 35.20 ± 14.52) completed an online survey. Participants were presented with a vignette depicting a woman with BED and obesity, followed by questionnaires assessing BED recognition, stigma, and other respondent characteristics. Independent samples t-tests were performed to compare participants who recognized BED in the vignette with those who did not, based on sociodemographic characteristics (i.e., gender, age, income, education) and psychosocial variables (i.e., explicit and internalized weight bias, familiarity with BED). A multiple linear regression analysis was performed to identify the sociodemographic and psychosocial variables that were the most important in explaining the variance in stigma towards BED.

**Results:**

Results indicated that 33% of participants identified BED as the main problem in the vignette. Those who recognized BED were younger, more educated, more familiar with BED, and exhibited lower levels of stigma towards BED. The most significant factor in explaining stigma towards BED was explicit weight bias, particularly attributing obesity to a lack of willpower and disliking people with obesity. Identifying as a man and older age were also associated with greater stigma towards BED.

**Conclusion:**

The findings of the current study highlight the importance of comprehensive public awareness campaigns to improve recognition of BED and to reduce associated stigma.

## Abstract (plain language summary)

Binge eating disorder (BED) is the most common eating disorder but is still largely unrecognized and stigmatized among the general public. This study aimed to explore how well BED is recognized and the stigma associated with it in a community sample of 894 adults (88.6% women; mean age of 35.20). Participants completed an online survey with a vignette depicting a woman with BED and subsequent questionnaires. Around one third (33%) of participants identified BED as the main issue in the vignette. Those who recognized BED tended to be younger, more educated, more familiar with BED, and exhibited lower levels of stigma. Explicit weight bias, such as attributing obesity to a lack of willpower and disliking people with obesity, was the most significant factor in explaining stigma towards BED. Additionally, identifying as a man and being older were linked to greater stigma. These findings underscore the need for public awareness campaigns to improve BED recognition and reduce stigma.

## Background

Binge eating disorder (BED) is an eating disorder (ED) characterized by recurrent episodes (on average at least once a week for three months) of binge eating (i.e., episodes of objective overeating in a discrete period of time (e.g., within any 2-hour period) while experiencing a subjective loss of control during the eating) associated with marked distress regarding binge eating [1]. BED is a serious condition that is associated with elevated risk of medical and psychiatric comorbidities and reduced quality of life [2, 3]. It is highly associated with overweight and obesity, with individuals living with BED presenting with overweight or obesity in 31.8% and 41.7% of cases, respectively [4]. The co-occurrence of BED and obesity would increase the burden of BED on health [5]. Although BED is the most prevalent ED with a lifetime prevalence of 2.8% [6], it still lacks recognition and awareness (i.e., inability to identify the disorder) from the general public globally [7].

Individuals with BED, like those living with other mental health disorders [8], are confronted with social stigma (i.e., being socially devalued due to an attribute that is deeply discrediting [9]) for their condition [10–12]. Examples of stigmatizing beliefs held are that people with BED are responsible for their condition and that they lack willpower, self-control, and self-discipline [10, 13]. Individuals with BED have also been characterized by less favorable personality traits (such as being perceived as weak, lazy, and careless) compared to individuals without BED [10]. Similar negative attributions are known to be made towards individuals living with obesity [14, 15] who frequently report experiencing high levels of weight stigma. Weight stigma can be defined as social devaluation due to excess body weight that can result in negative attitudes and discrimination [16]. Individuals diagnosed with BED reported greater lifetime exposure to experiences of weight stigma [17], which have consistently been shown to contribute to binge eating [18]. A vignette study showed that people living with both BED and obesity face increased stigma compared to people with obesity alone [19]. However, two other vignette studies found the opposite, i.e., no additive effect between BED and obesity stigma [12, 20].

The number of studies on BED recognition in the general population has increased in recent years [7, 21, 22]. Most of them used a case vignette methodology (e.g [21–23]), in which participants are presented with a vignette depicting a character with BED and are prompted to answer questions regarding this character. Among the adult general population, only three studies on BED recognition were conducted so far to our knowledge. In 2008, Mond and Hay (2008) reported that 11.7% of their Australian sample (*n* = 1031) correctly recognized BED using a vignette depicting a woman living with both BED and obesity [23]. Most participants rather identified depression (28.5%) or low self-esteem (28.3%) as the main problem of the character. Hollett and colleagues (2023) found, in a Canadian adult community sample (*n* = 235), that more than the half of participants (58.7%) correctly identified BED as the character’s main problem in a vignette depicting a woman presenting with BED without mention of body weight [21]. They also reported that younger participants were more effective at identifying BED compared to older participants, and that there was no significant difference between men and women in their ability to recognize BED. However, more than half of participants (54.9%) reported having experienced a similar problem to the character depicted in the vignette in the past, and almost a third (32.9%) believed currently having a similar problem. As stressed by the authors, this limits the generalizability of their results, as people living with eating problems may be better informed about eating disorders than a randomly selected sample [21]. Recently, Sala and colleagues (2024) conducted a vignette study with 276 community adults to examine whether the recognition of different EDs (including BED) varied depending on the gender of the individual depicted in the vignette [22]. Participants, irrespective of the gender condition, were more likely to recognize a problem in the anorexia nervosa (AN) and bulimia nervosa (BN) vignettes compared to the BED vignette. The recognition of BED as an ED did not vary significantly across gender conditions, suggesting that the gender of the target did not affect participants’ ability to identify BED [22].

As mentioned earlier, both BED and obesity are stigmatized conditions [10, 15]. The inconsistency between available results regarding the potential compounded effect of BED and weight stigma [12, 19, 20] stresses the need for a better understanding of how BED stigma and weight stigma are associated. Up to now, studies have focused on comparison of respondent stigmatizing attitudes depending on the presence or absence of BED and obesity in a vignette. However, no studies examined how explicit weight bias (i.e., negative attitudes about people because of their body weight) and internalized weight bias (i.e., application of negative societal weight-related stereotypes to oneself and self-stigmatization) may contribute to the explanation of stigmatizing attitudes towards people living with BED and obesity. This question is of particular interest as an important proportion of people living with BED also present with obesity [5] and both these conditions are subject to attitudes that stem from a common belief, that is attributing the conditions to a lack of willpower and personal weakness [10, 15, 20, 24]. Focusing on individuals living with both BED and obesity is important, as they face not only the distress of losing control over their eating but also the stigma and physical health issues associated with obesity [5, 18].

From all available studies on recognition and stigma of BED, a very few examined the associations between respondents’ personal characteristics and their knowledge and attitudes towards BED specifically. To our knowledge, only gender has been studied, showing that being a man was associated with higher levels of BED stigma [19, 25]. Given the limited literature on other respondents’ characteristics, drawing on studies that have explored the stigma surrounding other EDs, such as AN and BN, can provide valuable insights. For instance, it was shown that younger individuals, people with low income and those with lower education levels were more likely to express stigma towards AN and BN [10]. Studies on AN and BN stigma have yielded mixed results regarding the level of familiarity with these EDs. Some showed that unfamiliarity with AN and BN was associated with greater stigmatizing attitudes towards individuals with AN and BN [26, 27], while others found no association between the level of familiarity and AN and BN stigma [28–31]. Regarding respondent’s body weight, the only available study was conducted among undergraduates and suggested that those categorized as underweight reported lower levels of stigma towards AN and BN [29]. Overall, little is known regarding respondents’ characteristics that are associated with both recognition and stigmatization of BED specifically. It is important to deepen our understanding on this, as it may help in directing prevention and intervention efforts to improve the recognition of BED and to reduce the stigma associated with it. Enhancing BED recognition in the general population could have several benefits, including earlier identification of the disorder, which enables individuals to seek treatment sooner, increasing social support for those living with BED, and informing public health policies related to BED [32].

The present study had three aims. Using a vignette design: (1) to examine BED recognition in a vignette featuring a woman with both BED and obesity; (2) to compare participants who recognize BED diagnosis and those who do not according to different sociodemographic characteristics (age, gender, body mass index (BMI), income, education level) and psychosocial variables (explicit and internalized weight bias, familiarity with BED); (3) to examine the contribution of sociodemographic characteristics (age, gender, BMI, income, education) and psychosocial variables (explicit and internalized weight bias, familiarity with BED) in explaining stigmatizing attitudes towards BED.

Based on the only other Canadian study examining BED recognition in adults [21], it was hypothesized that most participants (more than the half) would recognize BED as the character’s main problem in the vignette. Furthermore, it was hypothesized that women would recognize BED in greater proportion than men, and that younger people would recognize BED in a greater proportion than older individuals. Regarding stigma towards BED, based on previous research on BED and other EDs stigma, it was expected that being a man [33] and being older [34] would be associated with higher levels of stigma towards BED. No hypotheses were formulated concerning the relationships between other respondent characteristics (i.e., BMI, income, and education level), explicit and internalized weight bias, and stigma towards BED due to limited literature.

## Methods

### Participants and procedure

A survey-vignette design was used. The sample consisted of adults recruited online from April to December 2019. To be included in the study, participants had to meet the following criteria: (1) being 18 years of age or older, (2) being a resident of the province of Quebec (Canada), and (3) being able to read and understand written material. Participants were recruited through different channels, i.e., Facebook posts, email lists of employees and students from three Universities in the province of Quebec with which the main researcher (CB) had connections, email lists of research volunteers from the general population, and email lists from research groups (composed of university students and researchers) and community organizations (reaching general population). The recruitment advertisement did not specify that the research focussed on BED recognition and stigma, it was entitled “Attitudes and knowledge related to weight and eating”. The recruitment link that was included in the advertisement directed participants to a secured survey website (*LimeSurvey*) on which the survey was hosted. Participants were first asked to answer a few questions to confirm their eligibility for the study and then, eligible participants were directed to the consent form previously approved by the Research Ethics Board of Laval University. Participants who consented were then invited to complete the survey, including a vignette depicting a woman presenting with BED and obesity, along with questions designed to assess respondents’ recognition of BED. After answering the questions accompanying the vignette (see section *Measures*), participants were informed that the character was living with BED and were provided with a brief description of the disorder. Participants were then asked to answer study questionnaires. Those who completed the survey and agreed to provide their contact information were entered into a lottery draw of six $50 gift cards to an online store.

### Measures

#### Vignette

A vignette describing the fictional case of a woman (Emily) living with both BED and obesity was presented to participants (Box 1). This vignette was previously used in past studies assessing recognition and attitudes towards BED [20, 35]. Following the reading of the vignette, participants were asked, “What would Emily’s main problem be?“. The following options were provided: “Bulimia nervosa”, “Anorexia nervosa”, “Binge eating disorder “, “Obesity”, “Lack of willpower and self-control”, “Loneliness”, “Low self-esteem/lack of self-confidence”, “Depression”, “Anxiety disorder/problem”, “No real problem”, and “I have no idea” (this question was originally used by Anderson et al., 2016 [36]). Participants were allowed to choose one option or more and were asked to rank their choices (if more than one) from most to least important.

**Table:** 

Box 1. Case Vignette	
Emily is a 19-year-old second-year art student. Emily has been overweight since she was an adolescent but in recent years this has increased to the point where her BMI is 35. Over the years Emily has tried a number of diets and healthy eating plans; however, she has never stayed with the recommendations for very long. Emily lives by herself and often feels lonely; to counteract these feelings Emily likes to ‘‘treat’’ herself with luxurious foods such as chocolate and cheesecake. Emily’s diet is generally regular, with three meals a day, and it contains a wide variety of foods. When Emily gets home from school she often goes to the fridge for a small snack; however, Emily finds that after eating the snack she is unable to control or stop her eating and continues to eat a large amount of food for a period of approximately two hours. She may binge eat, for example, on two slices of cheesecake, a bag of cookies, a jam sandwich and three glasses of milk in one sitting. Later in the evening she will eat dinner and sometimes she loses control with this also and eats the extra helping that she was planning to save for the next day. Emily feels uncomfortably full later at night and guilt and sadness after she has eaten like this, however she does not engage in any compensatory behaviors (e.g. purging). She has engaged in this binge-eating cycle twice a week for the past six months. Emily is very distressed by her eating behaviors, however has never disclosed this issue to anyone.	

#### Sociodemographic characteristics

Sociodemographic information of the participants, such as their gender, age, ethnicity, income, and education, was collected using a questionnaire created for this study.

#### Body mass index (BMI)

Participants provided self-reported measures of their weight and height. These measurements were converted to kilograms and meters, respectively, to compute participants’ BMI (kg/m^2^).

#### Explicit weight bias

The 13-item Anti-Fat Attitudes (AFA) questionnaire was used to assess explicit weight bias [37]. The AFA includes three subscales, Dislike, Fear of Fat, and Willpower. The Dislike subscale (7 items) assesses dislike of people with obesity (e.g., “I really dislike fat people.“). The Fear of Fat subscale (3 items) assesses concerns about one’s own weight and body shape (e.g., “One of the worst things that could happen to me would be to gain 25 pounds.“). The Willpower subscale (3 items) assesses beliefs that having overweight is a matter of willpower and self-control (e.g., “Some people are fat because they have no willpower.“). Items are rated using a 10-point Likert-type scale ranging from 0 “very strongly disagree” to 9 “very strongly agree”. Each subscale was scored by summing all the items. Higher scores reflect a higher level of explicit weight bias. Given that the Fear of Fat subscale assesses one’s concerns about gaining weight rather than prejudice or negative attitudes towards people with obesity [38], only the Dislike and Willpower subscales were utilized to assess explicit weight bias in the present study. The AFA have shown good psychometric qualities, including satisfactory construct validity and internal consistency [37]. In the current sample, the Cronbach’s alphas for the subscales were 0.80 for Dislike and 0.83 for Willpower.

#### Internalized weight bias

The Weight Bias Internalization Scale- Modified (WBIS-M) was used to assess the degree to which people apply negative weight-related stereotypes to themselves and self-devaluates because of body weight [39]. The modified version of the WBIS [40] was used in this study as this version is suitable for people across the BMI spectrum. The scale contains 11 items (e.g., “I hate myself for my weight,“) answered on a 7-point Likert scale ranging from “strongly disagree” to “strongly agree.” A total mean score can be obtained by averaging all the items. Higher scores reflect higher levels of internalized weight bias. The modified version of the WBIS has shown good psychometric properties, including a high internal consistency and good construct validity [39]. In the present study, the Cronbach’s alpha was 0.88.

#### Familiarity with BED

To assess the level of familiarity of participants with BED, the Level of Contact Report [41] was administered. The original measure described various levels of contact with mental illness in general [41], and was later adapted for AN [42–44]. The version used in the current study was adapted from the AN version from Crisafulli [43] to apply to BED. For each item, the word “Anorexia Nervosa” was replaced by “Binge eating disorder”. The scale contains 12 statements describing different levels of familiarity with BED, ranging from low (e.g., “I have never seen a person who I know had BED”), medium (e.g., “I have worked with a person who has/had BED”), to high (e.g., " I have binge eating disorder”). The participant was asked to select the statements that best represented their situation. The final score is the value of the rank of the most intimate item endorsed by the participant, so a higher score reflects greater familiarity with BED. This instrument has been used in several studies with different mental health disorders [45, 46], including AN [42, 43] and EDs more broadly [44, 47].

#### Stigma towards BED

The Eating Disorder Stigma Scale (EDSS) was used to assess stigma towards BED [48]. The scale was originally developed to assess stigma towards AN [48]. This questionnaire was adapted to the context of the current study to evaluate stigma towards BED specifically. Participants were asked to indicate their level of agreement with various statements about individuals with BED (with “AN” from the original version replaced by “BED” in both the questionnaire instructions and items). The subscale “Selfish/Vain” (e.g., “People with AN are obsessed with looking like supermodels”) used in the original AN version was removed since this type of prejudice is not or rarely conveyed towards people with BED. Therefore, the BED version of the scale contains 14 items rated on a 5-point Likert-type scale ranging from 1 “strongly disagree” to 5 “strongly agree” divided in three subscales: Trivial, Weakness and Blame. The Trivial subscale (5 items) assesses the meaninglessness of the disorder (*e.g*., “BED is not as serious as other mental illnesses”). The Weakness subscale (5 items) assesses beliefs that people with BED are weak and that they do not take power over their own behaviours (e.g., “People with BED are pathetic for not being able to control their ED”). The Blame subscale (4 items) assesses beliefs that people with BED are responsible for their condition. (e.g., “People with BED are responsible for their disorder”). A total EDSS score can be obtained by summing all items. Higher scores reflect higher levels of stigma towards BED. This tool is one of the most widely used in the assessment of AN stigma and demonstrated good psychometric qualities in past studies [42, 48]. In the present sample, the Cronbach’s alpha was .86.

### Statistical analyses

Analyses were performed using IBM SPSS Statistics-29 software package. All variables’ distribution were inspected for normality and outliers. A log transformation was applied to EDSS scores (stigma towards BED) to correct for a positive skew. Participants who had missing data were excluded solely from specific analyses in which pertinent items were absent (listwise deletion method).

In the first set of analyses, the sample was divided in two groups (1) people who recognized BED in the vignette and (2) people who did not. The first group included participants who identified BED as the character’s main problem in the vignette or as a second one, if obesity was identified as the first problem. Indeed, as both conditions were depicted in the vignette, the identification of BED and obesity as the two main problems was considered correct. All other participants were placed in the second group (i.e., people who did not recognize BED). Frequency analyses were performed to determine the percentage of respondents who correctly recognized BED (group 1) and to identify which problems were more frequently endorsed by the respondents who did not recognize BED (group 2) as the main problem in the vignette. Afterward, both groups were compared based on sociodemographic characteristics (i.e., gender, age, income, education, and BMI) and psychosocial variables (i.e., explicit and internalized weight bias, familiarity with BED) with independent samples t-tests and a Chi-square test of independence (for gender).

Among the entire sample, correlation analyses were performed to examine the associations between respondent characteristics and stigma towards BED. Then, to identify variables that were the most important in explaining the variance in stigma towards BED, a multiple linear regression analysis was performed. Only variables that were significantly correlated with stigma towards BED were included as predictors in the regression.

## Results

A total of 1,730 participants showed interest in the study by clicking on the link to get the survey. Data were excluded from respondents who did not reach the section of the questionnaire related to the vignette (*n* = 836). Therefore, the final sample comprised 894 participants. A priori power analysis indicated that our sample size was sufficient to detect small effects with 80% power and an alpha of 0.05 across the planned analyses. Age ranged from 18 to 86 years. Study participant characteristics are presented in Table 1 and descriptive statistics for study variables are presented in Table 2. Based on the Level of Contact Report, which measures levels of familiarity with BED—with the highest level corresponding to having BED—2.4% (*n* = 21) of the sample reported having BED.

**Table 1:** Sociodemographic characteristics of the sample (*n* = 894)

	M	SD
Age (*n* = 894)	35.20	14.52
BMI (kg/m²; *n* = 883)	25.86	6.38
	n	%
Gender (*n* = 890)		
Men	101	11.4
Women	785	88.6
Ethnicity (*n* = 875)		
White	848	96.9
Non-White	27	3.1
Familial income (CAD$) ^a^ (*n* = 823)		
0$ − 9 999$	110	13.4
10 000$ − 19 999$	130	15.8
20 000$ − 39 999$	136	16.5
40 000$ − 59 999$	117	14.2
60 000$ − 79 999$	91	11.1
80 000$ − 99 999$	72	8.7
100 000$ − 149 999$	119	14.5
150,000 or more	48	5.8
Education (*n* = 893)		
No schooling or incomplete primary education	1	0.1
Primary school	2	0.2
High school	93	10.4
College education ^b^	310	34.7
Undergraduate education	306	34.3
Master’s degree	156	17.5
Doctoral degree	25	2.8
BMI group ^c^ (*n* = 883)		
Underweight	37	4.2
Normal weight	441	49.9
Overweight	235	26.6
Obesity	170	19.3

Note. BMI = body mass index

^a^ The average familial income in the province of Quebec was 89,400$CAD at the time the study was conducted [49]

^b^ College refers to preuniversity or technical education

^c^ According to the WHO categorization: Underweight: BMI < 18.5; Normal weight: BMI between 18.5 and 24.9; Overweight: BMI between 25 and 29.9; Obesity: BMI ≥ 30

**Table 2:** Descriptive statistics for study variables (*n* = 894)

	M	SD
Stigma towards BED (*n* = 841)	1.51	0.50
Internalized weight bias (*n* = 894)	2.67	1.54
Explicit weight bias		
Dislike (*n* = 894)	1.41	1.33
Willpower (*n* = 894)	3.15	2.29
Familiarity with BED (*n* = 868)	3.76	3.30

Note. Stigma towards BED was assessed by an adapted version of the Eating Disorder Stigma Scale for BED, internalized weight bias by the Modified Weight Bias Internalization Scale, explicit weight bias by the Anti-Fat Attitudes questionnaire, and familiarity with BED by an adapted version of the Level of Contact Report

### Recognition of BED

According to the vignette, 33% (n = 295) of the sample correctly identified BED as Emily’ main problem. The other most selected responses were, in order of importance, loneliness (19.7%), BN (12.9%), and low self-esteem/lack of self-confidence (12.2%) as the character’s main problem. Only 4.6% of participants identified obesity as the main problem (without concurrently identifying BED).

Group comparison t-tests on sociodemographic and psychosocial variables between participants who recognized BED and those who did not are presented in Table 3. Overall, those who recognized BED were younger, more educated, more familiar with BED, and exhibited lower levels of stigma towards BED compared to those who did not recognize BED. For gender, the Chi-square test indicated that participants in the two groups did not differ on gender, χ^2^ (1, *N* = 886) = 2.61, *p* = .106. Women and men recognized BED in similar proportions.

**Table 3:** *C*omparisons of respondents’ characteristics depending on BED recognition (*n* = 894)

	Participants who recognized BED (*n* = 295)		Participants who did not recognize BED (*n* = 599)		t	*p*
	M	SD	M	SD		
Age	31.22	10.86	37.16	15.66	**5.48**	**< 0.001**
BMI (kg/m²)	25.43	6.51	26.07	6.31	1.41	0.158
Income	3.98	2.19	4.12	2.16	0.86	0.391
Education	4.83	0.94	4.58	1.00	**-3.49**	**< 0.001**
Internalized weight bias	2.72	1.60	2.65	1.51	-0.62	0.539
Explicit weight bias						
Dislike	1.44	1.39	1.39	1.30	-0.53	0.598
Willpower	3.00	2.25	3.22	2.31	1.34	0.181
Familiarity with BED	4.67	3.55	3.31	3.08	**-5.83**	**< 0.001**
Stigma towards BED	1.43	0.48	1.55	0.51	**3.37**	**< 0.001**

Note. Stigma towards BED was assessed by an adapted version of the Eating Disorder Stigma Scale for BED, internalized weight bias by the Modified Weight Bias Internalization Scale, explicit weight bias by the Anti-Fat Attitudes questionnaire, and familiarity with BED by an adapted version of the Level of Contact Report. BMI = body mass index

### Stigma towards BED

Correlational analyses between stigma towards BED and sociodemographic and psychosocial variables are presented in Table 4. Overall, identifying as a man, being older, reporting higher income, as well as higher levels of internalized and explicit weight bias (both Dislike and Willpower) were significantly associated with higher levels of stigma towards BED. Only the variables that were significantly associated with the stigma towards BED were entered in the regression analysis, namely gender, age, income, internalized and explicit weight bias.

**Table 4:** Bivariate correlations between study variables (*n* = 894)

	1	2	3	4	5	6	7	8	9	10
1. Stigma towards BED	-									
2. Women	− 0.172*	-								
3. Age	0.165**	− 0.127**	-							
4. BMI (kg/m²)	0.044	− 0.005	0.313**	-						
5. Income	0.083*	0.010	0.430**	0.163**	-					
6. Education	− 0.026	− 0.086*	0.142**	− 0.097**	0.274**	-				
7. Internalized weight bias	0.073*	0.121*	− 0.084*	0.361**	− 0.013	− 0.196**	-			
8. Explicit weight bias- Dislike	0.385**	− 0.078*	− 0.037	− 0.132**	− 0.038	− 0.013	0.203**	-		
9. Explicit weight bias- Willpower	0.444**	− 0.045	− 0.009	− 0.049	0.117**	− 0.055	0.131**	0.489**	-	
10. Familiarity with BED	− 0.011	0.019	− 0.08*	0.042	− 0.025	0.023	0.099	0.003	− 0.057	-

Note. Stigma towards BED was assessed by an adapted version of the Eating Disorder Stigma Scale for BED, internalized weight bias by the Modified Weight Bias Internalization Scale, explicit weight bias by the Anti-Fat Attitudes questionnaire, and familiarity with BED by an adapted version of the Level of Contact Report. BMI = body mass index

*p < .05 **p < .01

The results of the multiple linear regression are presented in Table 5. The overall regression model was significant (F (6, 764) = 53.28, *p* < .01) and explained 29% of variance in stigma towards BED (R^2^ = 0.29). Explicit weight bias explained the most variance in stigma towards BED followed by age and gender. Higher scores on the Dislike and Fear of fat subscales, as well as identifying as a man and older age, were associated with higher levels of stigma towards BED.

**Table 5:** Linear regression analysis predicting stigma towards BED with respondents’ sociodemographic and psychosocial characteristics (*n* = 765)

	B	ß	t	*p*
Women	**-0.05**	**-0.12**	**-3.73**	**< 0.001**
Age	**0.00**	**0.17**	**4.99**	**< 0.001**
Income	-0.00	-0.03	-0.76	0.446
Internalized weight bias	0.00	0.03	0.84	0.400
Explicit weight bias				
Dislike	**0.02**	**0.22**	**6.21**	**< 0.001**
Willpower	**0.02**	**0.35**	**9.80**	**< 0.001**

Note. Stigma towards BED was assessed by an adapted version of the Eating Disorder Stigma Scale for BED, internalized weight bias by the Modified Weight Bias Internalization Scale, and explicit weight bias by the Anti-Fat Attitudes questionnaire

## Discussion

The objectives of the present study were to examine BED recognition in a community sample of Canadian adults using a vignette featuring a woman with both BED and obesity and to compare participants who recognized BED diagnosis with those who did not, based on various sociodemographic characteristics and psychosocial variables. The study also aimed to examine the contribution of sociodemographic characteristics and psychosocial variables in explaining stigma towards BED. Overall, the results showed that 33% of participants recognized BED as the character’s main problem in the vignette. Participants who recognized BED were significantly younger, had a higher education level, were more familiar with BED, and showed lower levels of stigma towards BED. Furthermore, explicit weight bias (i.e., disliking people with obesity and believing obesity is due to a lack of willpower) were the most important variables in explaining stigma towards BED. Identifying as a man and older age were also significantly associated with greater stigma towards BED. This study is amongst the first to examine both BED recognition and stigma in an adult sample, and to explore the contribution of explicit and internalized weight bias in explaining stigma towards BED.

Among the present convenience community sample of Canadian adults, one third (33%) recognized BED as the main problem depicted in the vignette. This suggests that there is room for improvement in the general population’s knowledge regarding BED. The proportion of participants recognizing BED is smaller than what was expected. Hollett and colleagues (2023) recently found, in a convenience sample of Canadian adults from the community, that 58.7% of their participants recognized BED [21]. The discrepancy between these results and ours could potentially be related to the sample composition and to the vignette description. First, more than half of Hollett and colleagues’convenience community sample (54.9%) reported having experienced a similar problem to the character in the vignette in the past, and almost a third (32.9%) believed currently having a similar problem. In contrast, only 2.4% of participants in our sample reported having BED. This might result in Hollett’s participants having significant knowledge about EDs, which may have inflated their BED recognition rate. Future studies should use representative samples of the population in terms of gender, age, ethnic origin, and geography for better generalizability. Regarding vignette description, Hollett and colleagues (2023) did not include information about character’s body size in their vignette [21], while we did mention that the character was struggling with weight issues and provided specific information about her BMI, which placed her in the obesity category. This difference may have influenced the results. However, only 4.6% of our sample identified obesity as the main problem (without concurrently identifying BED). Although this might suggest that the presence of obesity as a characteristic of the character in our vignette did not lower the recognition rate of BED in our study, further research is required to fully understand the influence of weight status on people’s perceptions of BED. Participants who did not recognize BED mainly identified loneliness (19.7%), BN (12.9%), and low self-esteem/lack of self-confidence (12.2%) as the character’s main problem. This is in line with Mond and Hay’s (2008) study, which found that among a community sample of individuals aged 15 to 94 years, depression (28.5%) and self-esteem/self-confidence issues (28.3%) were most often identified by participants as the main problem of the character [23]. It suggests that education is needed to improve understanding that BED is a serious and complex mental health disorder, which is not simply a matter of loneliness or low self-esteem.

Comparisons of participants according to BED recognition status showed that those who correctly identified BED were significantly younger, which is in line with our hypothesis and previous studies [21]. Participants who recognized BED also had higher education levels. To our knowledge, this is the first time the relationship between participants’ education level and their ability to recognize BED has been examined; previous studies have only explored the association between education and beliefs about treatment, rather than BED recognition itself [21, 23]. Contrary to our hypothesis, no differences were found according to gender; men and women recognized BED in similar proportions. Nonetheless, past studies have suggested that women have higher levels of knowledge regarding EDs [36, 50] and better mental health literacy than men [51, 52]. Given that men were underrepresented in the current study (11.4% of the sample), results regarding gender should be interpreted with caution and need to be replicated. Furthermore, participants who correctly identified BED had higher levels of familiarity with BED. To our knowledge, this is among the first study to investigate the association between BED recognition and familiarity with this disorder. Future studies are needed to confirm this finding. Finally, individuals who correctly identified BED as the main problem in the vignette had significantly lower levels of stigma towards BED compared to those who did not identify BED properly. This is in line with literature on other mental health disorders, showing that greater mental health literacy is associated with better attitudes towards mental health issues [53]. Finally, it is worth noting that no significant difference was found between individuals who correctly recognized BED and those who did not regarding their level of explicit and internalized weight bias. Hence, it appears that knowledge of BED is associated with the level of stigma a person holds towards this specific mental health disorder, but not towards obesity.

In terms of the contribution of respondents’ sociodemographic and psychosocial characteristics to explaining stigma towards BED, our findings revealed that the variables explaining the most variance in stigma towards BED were explicit weight biases. Specifically, believing that obesity is due to a lack of willpower was the most significant factor, followed by disliking people living with obesity. Internalized weight bias did not stand out as significantly associated with stigma towards BED. Therefore, it is having negative attitudes towards people with obesity, rather than applying these negative attitudes to oneself, that is associated with stigma towards individuals with BED. It is not surprising that obesity and BED stigma are related, considering that explicit weight bias has been associated with various other types of stigmatizing attitudes (e.g., racist and homophobic attitudes) [54]. Moreover, it is worth noting that the character in the current study’s vignette had both BED and obesity, as BED often presents concurrently with obesity in 41.7% of cases [4]. This may have prompted participants to think of individuals with BED as also having obesity, which could have affected their perception of BED when assessing stigma associated with it. The stigma towards BED and obesity arises from common beliefs regarding the controllability of these conditions, specifically the notion that individuals living with them lack self-control [55]. According to Crandall (2003), attributing internal controllability to a condition may contribute to its stigmatization [56]. Therefore, it is important to challenge common misconceptions that imply weight and eating behavior regulation are solely within an individual’s control, thus suggesting that conditions like obesity and BED can be easily modified with mere willpower. BED is a serious mental health disorder with a multifactorial etiology [57], similar to obesity as a chronic disease [58]. It is imperative for the public to have a better understanding of BED in order to diminish stigma surrounding this disorder.

In accordance with our hypotheses, identifying as a man as well as older age were associated with higher levels of stigma towards BED. This is in line with past studies on stigma towards EDs [10, 13]. BMI, income, and education levels did not significantly contribute to explaining the variance in stigma towards BED. To our knowledge, this is the first study to explore the associations between those respondents’ characteristics and stigma towards BED specifically, as previous studies rather focused on stigma towards AN or BN [10]. Future studies are needed to confirm the absence of a relationship between these variables. It might seem surprising that there was no association between familiarity with BED and stigma towards BED, considering that studies on different mental health disorders consistently showed that greater contact with a person having a mental health disorder tends to reduce stigma towards this disorder [59, 60]. Nonetheless, previous studies on AN or BN have reported no associations between familiarity with these conditions and their stigmatization among the general population [28–31]. More research is needed to confirm this absence of relationship between familiarity and stigma towards BED specifically.

A strength of the current study is that it has examined the associations between respondents’ sociodemographic and psychosocial characteristics, including explicit and internalized weight bias, and BED recognition and stigma. Identifying respondents’ characteristics that are linked with BED recognition and stigma is important, as they may indicate specific targets for health promotion initiatives and stigma reduction interventions [61]. The current findings underscore the need to improve knowledge and attitudes relating to BED among older individuals, men, and people with lower levels of education.

It is important to acknowledge the limitations of the present study. First, despite the large sample size and its composition of adults from various age groups (age ranged from 18 to 86), the sample was mostly composed of women (88.6%) and White individuals (96.9%), which limits the generalizability of the current findings. The utilization of only one vignette depicting a character with both BED and obesity prevents the differentiation of the impact of obesity’s presence or absence on BED recognition and stigma. Additionally, as the character in the vignette was a woman, it is impossible to determine the effect of the target’s gender on BED recognition and stigma. To overcome these limitations, future studies could benefit from using multiple vignettes, randomly assigning them to participants. This approach would enable researchers to vary the characteristics of the depicted characters, including presenting BED with or without obesity and varying the character’s gender. This would contribute to refining our understanding of individual characteristics associated with BED that may influence public perception towards people living with this condition.

## Conclusions

The findings of the present study suggest that recognition of BED could be generally improved among adults, given that only one-third of our sample recognized BED as the character’s main problem in the vignette. Older age, lower education levels, lower familiarity with BED, and higher levels of stigma towards BED were identified as characteristics associated with poorer recognition of BED, offering potential targets for health promotion initiatives. Regarding stigma towards BED, explicit weight bias, rather than internalized weight bias, emerged as the most significant factor in explaining stigma towards BED, followed by older age and male gender. This highlights the contribution of weight bias in understanding stigma towards BED, emphasizing the importance of considering the potential impact of a person’s weight, who lives with BED, on the stigma they may experience related to this mental health disorder. Finally, the findings of the present study underscore the necessity for comprehensive public awareness initiatives targeting enhanced understanding of BED among the general population. It is necessary not only to improve recognition of BED in the general population but also to dispel misconceptions surrounding it in order to reduce the stigma associated with this condition. Future studies are needed to explore whether addressing explicit weight bias could reduce stigmatizing attitudes towards individuals living with both BED and obesity.

## Data Availability

The datasets used and analysed during the current study are available from the corresponding author on reasonable request.

## Abbreviations

AFA: Anti-Fat Attitudes
AN: Anorexia nervosa
BED: Binge eating disorder
BMI: Body mass index
BN: Bulimia nervosa
EDs: Eating disorders
EDSS: Eating Disorder Stigma Scale
WBIS-M: Weight Bias Internalization Scale- Modified
WHO: World Health Organization
